# Effect of Ultrasound-Enhanced Transdermal Drug Delivery Efficiency of Nanoparticles and Brucine

**DOI:** 10.1155/2017/3273816

**Published:** 2017-12-04

**Authors:** Nongshan Zhang, Yiyun Wu, Runlin Xing, Bo Xu, Dai Guoliang, Peimin Wang

**Affiliations:** Affiliated Hospital of Nanjing University of Chinese Medicine, Nanjing 210029, China

## Abstract

Brucine is the active component in traditional Chinese medicine “Ma-Qian-Zi” (Strychnos nux-vomica Linn), with capabilities of analgesic, anti-inflammatory, anti-tumor and so on. It is crucial how to break through the impact of cuticle skin which reduces the penetration of drugs to improve drug transmission rate. The aim of this study is to improve the local drug concentration by using ultrasound. We used fresh porcine skin to study the effects of ultrasound on the transdermal absorption of brucine under the influence of various acoustic parameters, including frequency, amplitude and irradiation time. The transdermal conditions of yellow-green fluorescent nanoparticles and brucine in skin samples were observed by laser confocal microscopy and ultraviolet spectrophotometry. The results show that under ultrasonic conditions, the permeability of the skin to the fluorescent label and brucine (e.g., the depth and concentration of penetration) is increased compared to its passive diffusion permeability. The best ultrasound penetration can make the penetration depth of more than 110 microns, fluorescent nanoparticles and brucine concentration increased to 2-3 times. This work will provide supportive data on how the brucine is better used for transdermal drug delivery (TDD).

## 1. Introduction

Traditional Chinese medicine “Ma-Qian-Zi” is a dry, mature seed of Strychnos nux-vomica Linn. Traditional Chinese medicine believes that “Ma-Qian-Zi” has the capabilities of improving microcirculation, detumescence and relieving pain. Modern research shows that the main active ingredient is alkaloids, the content of about 5% of crude drugs, mainly brucine, strychnine and its nitrogen oxides, of which brucine 1% [1]. Brucine is a weakly basic indole alkaloid, molecular formula C_23_H_26_N_2_O_4_, molecular weight 394 kDa, which is white crystalline powder, slightly soluble in water, soluble in ether, chloroform, ethanol, methanol and other organic solvents and tastes bitter [2–4]. It has good analgesic, anti-inflammatory, anti-tumor and other effects confirmed by related studies [5, 6]. However, it can excite the central nervous system. Recently, it becomes a hot spot in clinic how to give its full play to biological activity while avoiding the central toxicity [7–9].

Up to now, studies of brucine are mainly focused on reducing the central toxicity by transdermal administration and improving the efficacy through a novel drug carrier [3, 10]. Transdermal drug delivery (TDD) has a great advantage over oral administration, which avoids digestion and absorption of the gastrointestinal tract and thus largely reduces drug toxicity and bypasses the first effect of drug absorption, effectively improve the local drug concentration. However, due to the skin of the stratum corneum on the absorption of drugs has a certain blocking effect, transdermal drug absorption efficiency is often not satisfactory [11]. Therefore, the study of transdermal absorption of drugs is committed to the development of efficient and safe penetration technology which attracts lots of research, including iontophoresis, electroporation, micro-needle, ultrasonic penetration [12, 13]. Ion introduction is a very effective method of transdermal administration, but the drug must be ionized before transdermal and the application is inconvenient [14, 15]. Electroporation through the short wavelength, high voltage pulse to improve the permeability of the skin which will cause damage and pain resulting in the application limited [16]. Ultrasound-facilitated TDD enhances drug transdermal transport for most drug molecules [17, 18]. At the same time, ultrasonic penetration avoids the injury of organism and is flexible with other transdermal penetration technology, becomes a new drug research hotspot through the field of new drug penetration [19].

Ultrasound on the drug transdermal absorption efficiency is mainly related to frequency, intensity, duty cycle, irradiation time [19]. The basic principle of ultrasonic penetration is thought to mainly include the thermal effect of ultrasonic energy absorption, the cavitation effect caused by the collapse and oscillation of cavitation cavities, and the cavitation effect is considered to be the main mechanism of ultrasonic penetration [20–22]. Although the cavitation effect is a violent process, studies have shown that the process does not cause significant or severe skin damage [23, 24]. Transdermal drug related research is the most concerned about the drug is insulin, small a molecule protein with molecular weight of about 5.6 kDa [25, 26]. Research reports in terms of high molecular weight drug molecules similar to brucine alkaloids remain lesser [27].

In this study, the detection of yellow-green fluorescent nanoparticles and conditions of brucine through porcine skin samples were examined in different ultrasonic parameter conditions (i.e. frequency, amplitude, and irradiation time). The results of this work will provide supportive parameters on how to better use ultrasound technology to promote the brucine drug transdermal absorption to increase the pesticide effect.

## 2. Material and Methods

### 2.1. Experimental Setup and Porcine Transdermal Model

In the present study, porcine skin was used as the transdermal model. Previous studies have shown that the thickness of the different layers of the porcine skin is similar to that of the human skin [28, 29]; the elasticity and cell structure are similar to those of the human skin; and the transmission speed of the sound is similar (porcine skin 1720 m/s, human skin 1498–1650 m/s). Fresh porcine's back skin (2 mm thick) was shaved, cut into a 25 square centimeters square piece and placed in 37°C of water to wash away the skin surface grease. Subsequently, the porcine skin samples were placed in the middle of the Franz diffusion pool and clamped with clips. The bottom of the diffusion pool is covered with sound-absorbing rubber to avoid ultrasonic reflection at the bottom of the tank.

**Figure 1:**
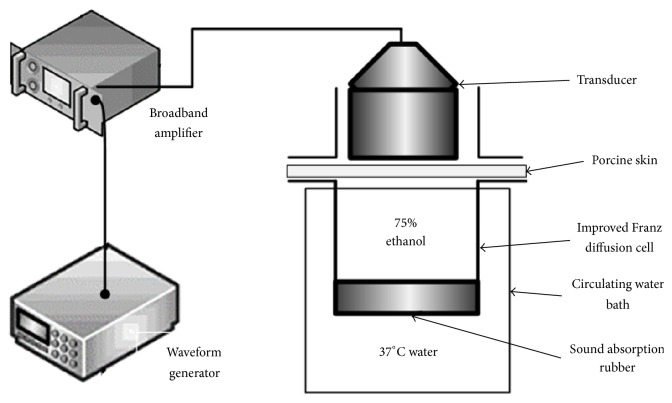
In vitro experimental system of transdermal model of porcine skin.

As shown in Figure 1, the entire system is consisted of a waveform generator (33250A, Agilent, CA, USA), a power amplifier (2200L, E & I, NY, USA), an ultrasonic transducer, a circulating water bath, and an improved Franz (Franz) diffusion pool. The Franz diffusion tank is fixed on a 37°C circular water bath by a frame.

### 2.2. Evaluation of Fluorescence Particle Permeability

Based on the methods mentioned in previous studies [30, 31], we evaluated the fluorescence article permeability. The yellow-green fluorescent nanoparticles have a diameter of 50 nm and a density of 1.05 g/cm^3^ (Fluoro-MaxTM G50, Thermo Fisher Scientific, MA, USA). The reagent was shaken before each experiment and 30 uL of the middle layer was added to 6 ml of water, which ensured that the final concentration of the mixture in the diffusion tank was 1 : 200.

Four different frequency and diameter ultrasonic transducers will be used in the experiment. (50 kHz/38 mm, 200 kHz/31 mm, 643.5 kHz/25.4 mm, and 1 MHz/12.7 mm). At each measurement, the transducer surface and the porcine skin samples were immersed in a diffusion cell, and the transducer was placed on the surface of the porcine skin by 0.5 mm. Each transducer was irradiated with ultrasound at 10% duty cycle and 100 Hz pulse repetition frequency for 10 minutes at four different pressure amplitudes (25 kPa, 50 kPa, 75 kPa, and 100 kPa), respectively. The in situ pressure was calibrated with a needle hydrophone (HNC-0100, Onda Corporation, Sunnyvale, CA, USA) controlled by a three-dimensional positioning system (Newport Irvine, CA, USA), which was determined by adjusting the input voltage by a single source transducer. The laser confocal microscopy can be used to identify the difference between samples with ultrasonic irradiation and the case where the skin samples without ultrasonic irradiation instead of naturally infiltrated for 10 minutes by the depth of penetration and the total amount of permeation.

### 2.3. Evaluation of Brucine Permeability

Extraction of the concentration of 50 ug/ml brucine (lot: 16042903, Chengdu Pfeiffer Biotechnology Co., Ltd., China) solution 6 ml into the diffusion tank above the porcine skin. The solution was 75% ethanol. The subsequent method is the same as the fluorescence particle permeability test described above. Collect 45 ml of the receiving solution. Repeat the experiment three times for each acoustic parameter, respectively. The most suitable ultrasonic frequency and intensity were chosen to perform a comparative experiment at different times (5 min, 10 min, 20 min, 40 min, 60 min) to determine the effect of different time on the infiltration of brucine. The brucine content in receiving solution was detected by high performance liquid chromatography (LC-MS/MS).

### 2.4. Measurement of Penetration Depth and Concentration of Fluorescent Nanoparticles

After completion of the permeation experiment, the mixture of fluorescent nanoparticles was poured out. 2 ml of degassed water was injected into the diffusion cell and gently shaken to remove residual fluorescent nanoparticles from the surface of the porcine skin. The penetration depth and concentration of fluorescent nanoparticles were measured by laser confocal microscopy (Revolution XD, Andor, UK). Repeat the experiment three times for each acoustic parameter, respectively. After each experiment, the porcine skin was cut along the *Z*-direction and sliced with a thickness of about 2 mm. The sections were gently tiled on a confocal microscope special dish (bottom glass thickness 0.13 mm) for laser confocal microscopy. Adjust the laser confocal microscope to the excitation wavelength of 488 nm, the cut porcine skin samples were placed in the focal plane near the surface, so that the penetration of fluorescent nano-particles was visible. The skin pieces did not not always adhere to the bottom of the culture dish, so the *Z* axis of the pieces in the field of view were extended by 10 times (DZ = 10 um), of which the maximum fluorescence projection intensity was determined. At the same time each position was photographed three times with a fluorescence microscope and averaged to remove noise. In this way, the depth and concentration of the nanoparticles in the cross section of the skin sample under different ultrasonic parameters can be observed by laser confocal microscopy.

### 2.5. Content Detection of Brucine in Receiving Solution by LC-MS/MS

Analysis were performed with Agilent HPLC 1200 system (Agilent, USA) consisting of a quat pump, an autosampler and an online degasser. The chromatographic separation was performed on an Phenomenex Gemini C18, 3 *μ*m particle size, 110 Å, 100 mm (length) × 2.0 mm (I.D.) reversed phase analytical column. The mobile phase consisted of methanol-0.1% formic acid (80 : 20;* v*/*v*) at a flow rate of 0.2 mL·min^−1^. The autosampler temperature was maintained at 4°C and the injection volume was 5 *μ*L. The total LC run time was 4 min with the column temperature kept at 35°C.

Detection of analytes was performed on API 4000 tandem quadrupole mass spectrometer (Applied Biosystems, USA) with an electrospray ionization (ESI) interface in positive ion mode. Multiple reaction monitoring (MRM) was used to monitor precursor to product ion transition of* m/z *395.1 → 324.3 for brucine and* m/z *356.2 → 192.2 for tetrahydropalmatine (internal standard, IS). The analytical data were processed using Analyst software (version 1.4.1, Applied Biosystems).

For analyses and IS the source parameters were ion spray voltage 4000 V, turbo heater temperature 400°C, collision activation dissociation 6 psi, curtain gas 20 psi. The compound dependent parameters like declustering potential and collision energy were optimized at 110 V and 45 V for brucine and 150 V and 40 V for IS, respectively. Quadrupole 1 and quadrupole 3 were maintained at unit resolution. Dwell time set was 150 ms for both the analyze and IS.

## 3. Results and Discussion

### 3.1. Fluorescence Nanoparticles Penetration Depth and Concentration

**Figure 2:**
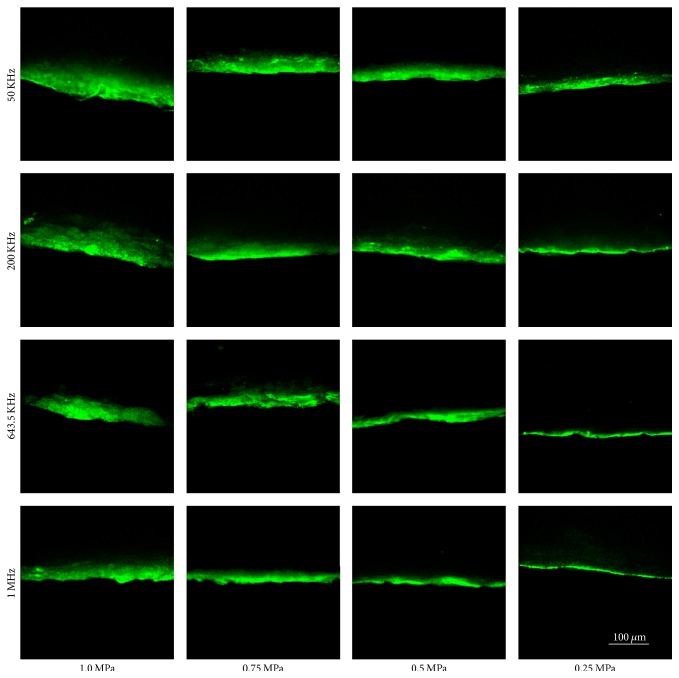
The confocal images of penetration depth at different frequencies and intensity under the influence of ultrasound alone for 10 mins.

Figure 2 shows the confocal microscopy of the depth and concentration of fluorescent nanoparticles in the skin samples after 10 minutes of ultrasonic irradiation at different frequencies and amplitudes. It is clearly observed that: (1) for a fixed frequency, the penetration depth and concentration decreases as the acoustic amplitude decreases; (2) for a fixed acoustic amplitude, the penetration depth and concentration decreases with the increase of the frequency. Permeability of the florescence nanoparticles reaches the peak at 50 kHz, 1.0 Mpa; whereas permeability of the florescence nanoparticles reaches the bottom at 1 MHz, 0.25 Mpa.

**Figure 3:**
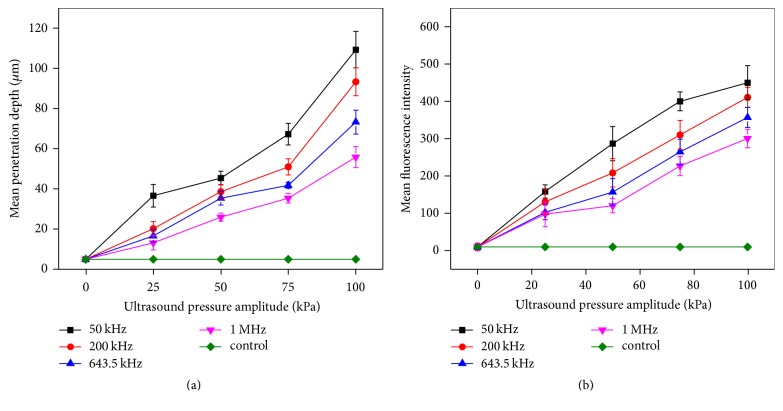
The curves of penetration amount and depth of fluorescent nanoparticles under the influence of ultrasound alone for 10 min. (a) The mean penetration depth; (b) the amount of permeation, the mean fluorescence intensity.

Compared with the control samples, the permeation concentrations were significantly increased at four different frequencies and intensities, as shown in Figure 3. The curve of the penetration depth versus intensity is plotted in Figure 3(a). In the case of the same intensity, the penetration depth was observed to decrease with the increase of frequency. As an example, under the action of 50 kHz ultrasound exposure, 100 kPa excitation caused the penetration depth of 110 um. In the case of the same frequency, the penetration depth increases with increasing strength.  Figure 3(b) shows the variation of average fluorescence intensity in per unit area with the increase in the intensity, showing a linear relationship change. In the case of the same intensity, the lower the frequency, the higher the average fluorescence intensity was observed; in the case of the same frequency, the average fluorescence intensity increases with the increase of intensity in strength.

As deep skin fluorescence intensity was weak, fluorescent particles were mostly concentrated in the skin surface (20–100 um) in the confocal microscopic image. Further experiments confirmed that that even further increasing the sound intensity, there was no significant increase in penetration depth and concentration.

### 3.2. Ultrasound Penetration of Brucine

**Figure 4:**
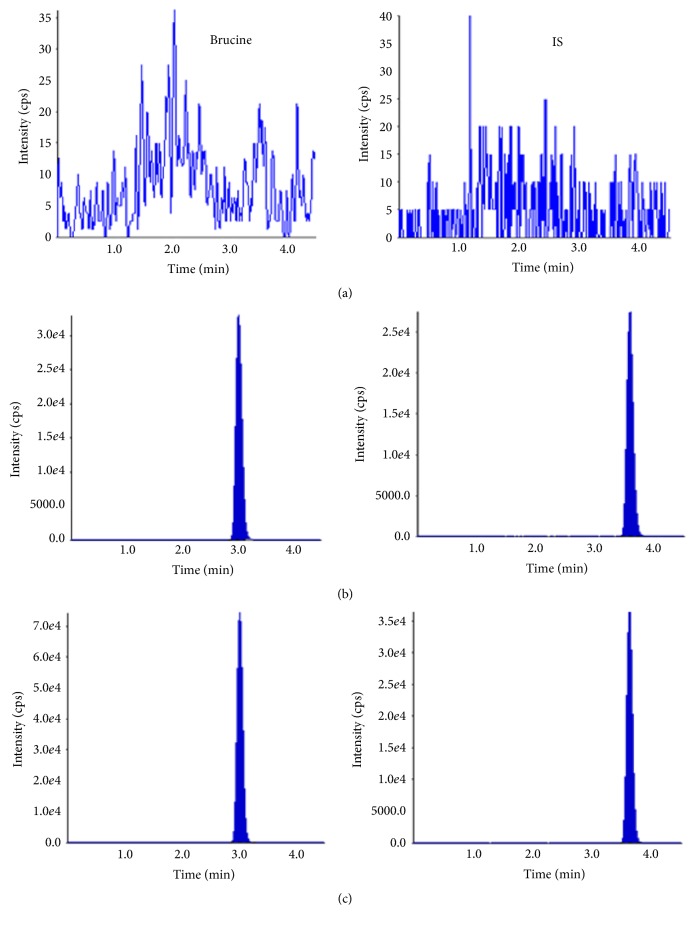
Chromatograms of brucine and tetrahydropalmatine. (a) a blank dialysate; (b) a blank dialysate spiked with standard solution; (c) the sample of dialysate.

The mass spectrometry detection gave very good selectivity for the brucine and IS.  Figure 4 shows the total ion chromatogram (TIC) of spiked dialysate with brucine (10 ng/mL) and IS (25 ng/mL), as well as TIC of dialysate sample at a frequency of 50 kHz and intensity of 75 kPa. The retention time was 3.0 min for brucine and 3.6 min for IS, respectively. No endogenous interference was detected in blank dialysate samples at the retention time of brucine and IS. No cross-talk peaks were observed among the brucine and IS after the analysis of the highest standard on calibration curve and working solution of IS. Typical equation of the calibration curve for brucine was: *y* = 0.4254*x* − 0.025 (*r* = 0.9946). The back-calculated concentrations at all point on the standard curve were within ±15% of the nominal concentrations. The lowest concentration at S/N ratios of 10 with the RSD < 20% was taken as lowest limit of quantification (LLOQ), and was found to be 0.5 ng/ml for brucine. For the study of brucine, the results are similar to those of fluorescent particles under ultrasound.

**Figure 5:**
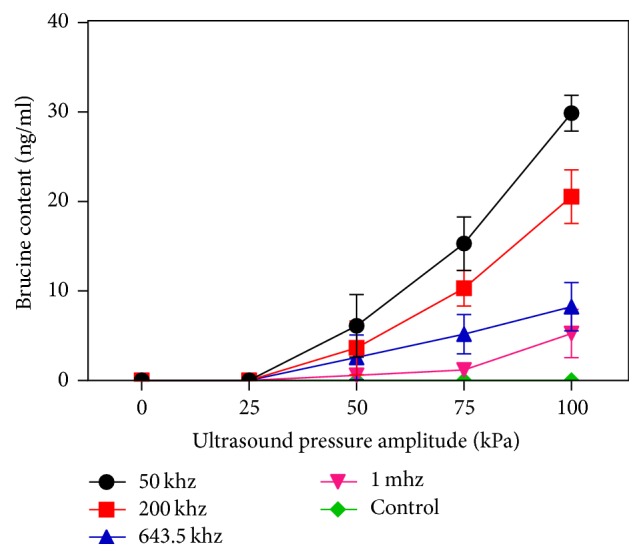
The contrast curves of Strychnine penetration under the influence of ultrasound for 10 mins.

Figure 5 shows the content of brucine transdermal receiving solution under ultrasound. Transparency is the largest for 50 kHz ultrasound at the amplitude of 100 kpa. When amplitude is fixed, low-frequency effect is relatively obvious, with the increase in frequency, transdermal reduced, the penetration is the largest for 50 khz. When frequency is fixed, the effect is obvious in high strength, with the increase of the intensity, the transdermal volume increases, the penetration is the largest at 100 kpa. The effect of all ultrasound groups was better than that of the control group.

**Figure 6:**
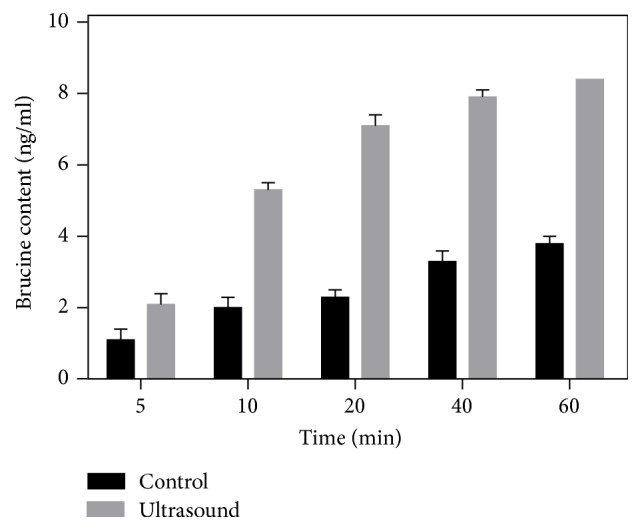
The variation curve of Strychnine penetration amount at a frequency of 50 kHz and a intensity of 100 kPa.

In order to determine the effect of ultrasonic action time on the depth of penetration and the amount of permeation, measurements at different time points were performed for 50 kHz ultrasound at the amplitude of 100 kPa.  Figure 6 shows the content of brucine in the receiving solution with the action time of the curve. With the increase of time, the amount of permeation is gradually increased. The change is obvious within 0–20 minutes, the trend of change of depth and concentration becomes gradual more than 20 minutes.

## 4. Conclusion

In this paper, the effects of different ultrasonic parameters (such as frequency, pressure amplitude and irradiation time) on the transdermal absorption efficiency of nanoparticles, brucine were studied. Under different conditions, the effect of ultrasound on the osmotic effect was assessed by measuring the penetration depth and effective permeation concentration of the fluorescent nanoparticles in the porcine skin samples. Differences of depth and concentration of brucine were determined by comparing the natural penetration and ultrasonic penetration. Under ultrasonic conditions, the permeability of the fluorescent particle marker and brucine (e.g., the depth and concentration of penetration) were both increased compared to its passive diffusion permeability. Low frequency wave ratio showed a better effect, with the strength and time to increase, the better the drug effect. The best ultrasound penetration can make the penetration depth of more than 110 microns, fluorescent nanoparticles and brucine base drug concentration increased to 2-3 times. We consider that the mechanism of the ultrasound-enhanced drug delivery is the microbubble cavitation, the surface of the porcine's skin may have obvious cavitations after ultrasound intervention. This indicates that the cavitation under ultrasound is an important mechanism to improve the permeability [32–37].

Brucine is made into transdermal dosage form, which can improve the local concentration of drugs and is beneficial to the treatment of local inflammation and pain, and can prevent the central toxic reaction. Our findings can serve as a supportive guide for transdermal administration. The development of transducers with improved performance to achieve more accurate ultrasonic intensity calibration, as well as optimization in the lag time and duty cycle and other parameters will be our follow-up further research direction. Since the side effect and toxicity of brucine are not quantified, more research should be undertaken in purification procedures to minimize the toxicity and maximize the advantages.
